# *VRN-1* gene- associated prerequisites of spring growth habit in wild tetraploid wheat *T. dicoccoides* and the diploid A genome species

**DOI:** 10.1186/s12870-015-0473-x

**Published:** 2015-03-31

**Authors:** Andrey B Shcherban, Kseniya V Strygina, Elena A Salina

**Affiliations:** Institute of Cytology and Genetics, Lavrentiev ave. 10, Novosibirsk, 630090 Russia

**Keywords:** Allelic diversity, Vernalization, *VRN-1* gene, Promoter, First intron, Tetraploid, Diploid, *Triticum*

## Abstract

**Background:**

In order to clarify the origin of spring growth habit in modern domesticated wheat, allelic variability of the *VRN-1* gene was investigated in a wide set of accessions of the wild tetraploid species *Triticum dicoccoides* (BBAA), together with diploid species *T. monococcum*, *T. boeoticum* and *T. urartu*, presumable donors of the A genome to polyploid wheats*.*

**Results:**

No significant variation was found at the *VRN-B1* locus of *T. dicoccoides*, whereas at *VRN-A1* a number of previously described alleles were found with small deletions in the promoter (*VRN-A1b*, *VRN-A1d*) or a large deletion in the first (1^st^) intron (*VRN-A1L*). The diploid A genome species were characterized by their own set of *VRN-1* alleles including previously described *VRN-A1f* and *VRN-A1h* alleles with deletions in the promoter region and the *VRN-A1ins* allele containing a 0.5 kb insertion in the 1^st^ intron. Based on the CAPS screening data, alleles *VRN-A1f* and *VRN-A1ins* were species-specific for *T. monococcum*, while allele *VRN-A1h* was specific for *T. boeoticum*. Different indels were revealed in both the promoter and 1^st^ intron of the recessive *VRN-A1u* allele providing specific identification of *T. urartu*, the proposed donor of the A genome to modern wheat. We found that alleles *VRN-A1b* and *VRN-A1h*, previously described as dominant, have either no or weak association with spring growth habit, while in some diploid accessions this habit was associated with the recessive *VRN-A1* allele.

**Conclusions:**

Spring growth habit in diploid wheats was only partially associated with indels in regulatory regions of the *VRN-1* gene. An exception is *T. monococcum* where dominant mutations in both the promoter region and, especially, the 1^st^ intron were selected during domestication resulting in a greater variety of spring forms. The wild tetraploid *T. dicoccoides* had a distinct set of *VRN-A1* alleles compared to the diploids in this study, indicating an independent origin of spring tetraploid forms that likely occurred after combining of diploid genomes. These alleles were subsequently inherited by cultivated polyploid (tetraploid and hexaploid) descendants.

**Electronic supplementary material:**

The online version of this article (doi:10.1186/s12870-015-0473-x) contains supplementary material, which is available to authorized users.

## Background

*Triticum dicoccoides* or *T. turgidum* ssp. dicoccoides Thell is considered to be the wild form of all domesticated emmer wheats [1]. The species has two homologous sets of chromosomes (BBAA) thought to have arisen from spontaneous hybridization between two diploid donor species. The donor of the B genome is unknown but was likely a species closely related to modern *Aegilops speltoides* Tausch (2n = 14; SS) [2-4]. Potential sources of the A genome are represented by three closely related species: *T. urartu* Tum. ex Gandil., *T. boeoticum* Boiss. and *T. monococcum* L. These species are separated by crossing barriers [5], and differ in their plant morphology [6] and biochemical and molecular marker loci [3,7,8]. *T. boeoticum* is considered to be the progenitor of cultivated diploid einkorn wheat, *T. monococcum. T. urartu* is considered as the donor of A genome to the wild tetraploid species: *T. dicoccoides* (BBAA) and *T. araraticum* (GGAA) [9], which was subsequently inherited by other important polyploid wheat species including *T. durum* (BBAA), *T. timopheevii* (GGAA), and common wheat *T. aestivum* (BBAADD). Despite many studies of *Triticum* domestication, employing morphological, cytological [6,10] and molecular marker analysis techniques [7,11-14], many questions remain unanswered with respect to the phylogenetic relationships between diploid wheat species and between diploids and their polyploid descendants.

Adaptability of polyploid wheat to a wide range of environments has been at least partially facilitated by allelic diversity in *VRN* genes regulating growth habit and flowering time [15]. Wheats are categorized into two major forms: those requiring vernalization or exposure to cold to accelerate flowering (winter forms) and those which do not require vernalization (spring forms). Vernalization prevents floral initiation during winter, thereby protecting sensitive floral meristems from freezing temperatures. In recent years, researchers have significantly advanced our understanding of the molecular mechanisms of vernalization in wheat. The genomic locations and sequences of three vernalization genes (*VRN-1*, *VRN-2* and *VRN-3*) responsible for spring/winter growth habit have been determined [16-18].

The *VRN-1* gene is proposed as the main initiating factor of the regulatory cascade initiating flowering [16,19]. *VRN-1* encodes a MADS-box transcription factor which controls the transition of the vegetative shoot apical meristem to the reproductive phase [20,21]. In vernalization-requiring cereals, *VRN-1* expression is induced by vernalization, with the level of expression being dependent on the length of cold exposure [22]. *VRN-1* downregulates the floral repressor *VRN-2*, and allows long-day induction of the floral activator *VRN-3* to accelerate subsequent stages of floral development [17,19].

Changes in the growth habit of winter wheat to spring wheat are primarily due to dominant mutations in regulatory regions (promoter or intron 1) of *VRN-1* [15]. *VRN-A1* alleles of the hexaploid or common wheat *T. aestivum* L. (2n = 42, BBAADD) and the tetraploid *T. turgidum* L. (2n = 28, BBAA), have so far been identified containing nucleotide deletions as well as insertions of mobile elements in both regions [23,24]. By contrast, dominant mutations in *VRN-B1* and *VRN-D1* genes are predominantly caused by large deletions in the 1^st^ intron [24]. Recently, a novel *VRN-B1* allele was identified as having a deletion coupled with sequence duplication within intron 1 [25-27].

Most of the wild *Triticeae* species have a winter growth habit, suggesting that the recessive *VRN-1* allele is the ancestral form. By contrast, there are many cultivated polyploid wheats with a spring growth habit and with at least one dominant *VRN-1* allele [28]. Reports demonstrate that different combinations of dominant and recessive *VRN-1* alleles (*VRN-1* genotype) significantly affect the time of flowering [29,30]. Spring growth habit determined by dominant *VRN-1* alleles could be either inherited from ancestral diploids or could result from selection of independent mutations appearing during the adaptation to different environments after domestication. To resolve this issue, a large scale screening of *VRN-1* polymorphisms among both diploid and polyploid wheat species is required. To date, a limited number of accessions of diploid and tetraploid progenitors of *T. aestivum* have been examined [16,23,24,31,32].

In the present study, molecular variability of *VRN-1* genes was analyzed based on a representative set of accessions of wild tetraploid wheat and diploid A genome species. The main objective was to identify and characterize different alleles of *VRN-1* genes and to analyze their distribution among accessions. We also examined the influence of different alleles and their combinations on vernalization requirement. This analysis sheds light on the evolution of *VRN-1* genes in diploids and during the first stages of wheat polyploidization. It contributes to our understanding of the phylogenetic relationships between diploid and polyploid wheat species and the evolutionary history of modern domesticated wheat.

## Results

### *VRN-1* allelic variability in wild tetraploid wheat *T. dicoccoides*

#### Promoter region

**Figure 1:**
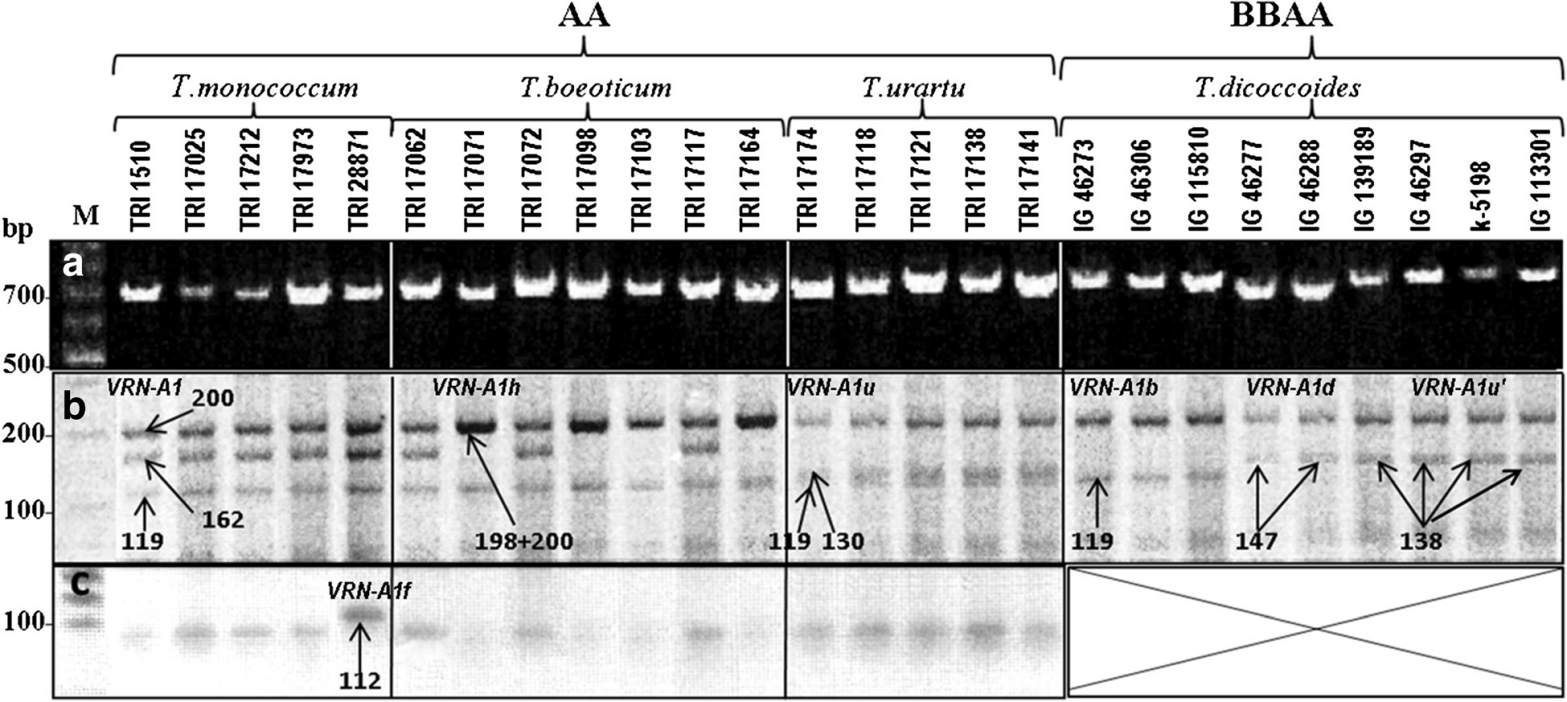
**PCR amplification of** ***VRN-1*** **promoter region. a**- PCR amplification with primers VrnA1F/Int1R.** b**- *Msp* I restriction digestion of corresponding PCR products. **c**- PCR amplification with primers VRN1F_F/VRN1F_R followed by *Taq* I restriction digestion. Accession numbers, species and genotypes are given at the top. The main restriction fragments more than 100 bp in length are indicated by arrows.

Vernalization sensitivity in tetraploid wheat *T. dicoccoides* is controlled by alleles at the 2 homoeologous loci, *VRN-A1* and *VRN-B1*. The specific primers Vrn1AF and Int1R were used in order to identify variation in the promoter region of *VRN-A1* locus as described by Yan et al. [23] (Table 1). Almost all of the 80 studied accessions of *T. dicoccoides* yielded a PCR product of approximately 0.7 kb (Figure 1a). To further analyse these PCR products, we digested them with a frequently-cutting restriction endonuclease *Msp* I. The restriction patterns could be divided into two types (Figure 1b). The first type, characteristic of the vast majority of the studied accessions, contained two major fragments of ~140 and 200 bp long. The second type was found in only 3 of the 80 *T. dicoccoides* accessions and was characterized by a fragment of approximately 120 bp in place of the 140 bp band. To further analyze allelic variation at the *VRN-A1* promoter, we selected a set of accessions representing both restriction patterns and sequenced the PCR products obtained with VrnA1F/Int1R primers (Table 2).

**Table 1:** **PCR markers for determining the presence of different alleles of** ***VRN-A1*** **,** ***VRN-B1*** **in diploid and polyploid wheats**

**PCR marker**	**Name**	**Primer (5′→3′)**	**Target allele(s)**	**Expected product size (bp)**	**Annealing temp. (ºC)**	**Reference.**
*VRN-A1* marker*	Vrn1AF	GAAAGGAAAAATTCTGCTCG	*VRN-A1a*	876 and 965	55.0	[16,23,31]
	Int1R	GCAGGAAATCGAAATCGAAG	*VRN-A1b*	694		
			*VRN-A1d*	662		
			*VRN-A1h*	684		
			*VRN-A1f*	703		
			*VRN-A1*	704		
			*VRN-A1u*	705		
			*VRN-A1u*	713		
*T. monococcum VRN-A1*	Indel(-)F	CGCTCTTATATTTGTTTACCAGGG	*VRN-A1*	1025	50.0	_
	Indel(-)R	GGGTCAACTATTCTGTGGAG	*VRN-A1u, VRN-A1u’*	no product		
*VRN-A1* deletion of 1.4 kb	Intr1/C/F	GCACTCCTAACCCACTAACC	*VRN-A1u, VRN-A1u’*	1068	56.0	[24]
	Intr1/AB/R	TCATCCATCATCAAGGCAAA				
*VRN-A1* insertion of 0.5 kb	Intr 1	ATCATCTTCTCCACCAAGGG	*VRN-A1ins*	1980	50.0	_
	Intr1insR	AATGAACAGCACGGAAACAG	*VRN-A1*	1476		
*VRN-A1* deletion of 7,2 kb	Ex1/C/F	GTTCTCCACCGAGTCATGGT	*VRN-A1L*	522	55.6	[24]
	Intr1/A/R3	AAGTAAGACAACACGAATGTGAGA				
*VRN-B1* marker*	P2	TCATGCACGCACACACGGTA	*VRN-B1*	814	55.0	[25]
	P5	GGCCAACCCTACACCCCAAG				
*VRN-B1* Non-deletion	Intr1/B/F	CAAGTGGAACGGTTAGGACA	*VRN-B1*	1149	56.4	[24]
	Intr1/B/R4	CAAATGAAAAGGAATGAGAGCA				

*These diagnostic markers detect allelic variation at the promoter regions. In other cases variation within intron 1 of corresponding genes is detected.

**Table 2:** **Allelic variants of** ***VRN-A1*** **gene in a selected set of accessions of diploid** ***Triticum*** **species and tetraploid** ***T. dicoccoides***

**Species (genome)**	**Accession number**	**Growth type (Winter/ Spring)**	***VRN-A1*** **structural forms** *			Alleles**	**GenBank Ac.N.**	**Homologous sequences from GenBank**
			**Promoter**	**Intron 1**				
				**Insertion**	**Deletions**			
				**0.5 kb**	**1.4 kb/7.2 kb**			
*T. monococcum* (AA)	TRI 1510	S	*VRN-A1*	*+*	*−/−*	*VRN-A1ins*	KM586653	AY188331
	TRI 17025	S	*VRN-A1*	*-*	*−/−*	*vrn-A1vrn2*	-//-	-//-
	TRI 17212	S	*VRN-A1*	*+*	*−/−*	*VRN-A1ins*	-//-	-//-
	TRI 17973	S	*VRN-A1*	*-*	*−/−*	*vrn-A1VRN-2*	-//-	-//-
	TRI 28871	S	*VRN-A1f*	*+*	*−/−*	*VRN-A1f VRN-A1ins*	KM586656	DQ146421
*T. boeoticum* (AA)	TRI 17062	W	*VRN-A1*	*-*	*−/−*	*vrn-A1*	KM586653	AY188331
	TRI 17071	S	*VRN-A1h*	*-*	*−/−*	*VRN-A1h*	KM586657	GQ451745
	TRI 17072	S	*VRN-A1*	*-*	*−/−*	*vrn-A1VRN-2*	KM586653	AY188331
	TRI 17098	S	*VRN-A1h*	*-*	*−/−*	*VRN-A1h*	KM586657	GQ451745
	TRI 17103	W	*VRN-A1h*	*-*	*−/−*	*VRN-A1h*	-//-	-//-
	TRI 17117	W	*VRN-A1*	*-*	*−/−*	*vrn-A1*	KM586653	AY188331
	TRI 17164	W	*VRN-A1h*	*-*	*−/−*	*VRN-A1h*	KM586657	GQ451745
*T. urartu* (AA)	TRI 17174	W	*VRN-A1u*	*-*	*+/−*	*vrn-A1u*	KM586659	GQ482970
	TRI 17118	W	*VRN-A1u*	*-*	*+/−*	*vrn-A1u*	-//-	-//-
	TRI 17121	W	*VRN-A1u*	*-*	*+/−*	*vrn-A1u*	-//-	-//-
	TRI 17138	W	*VRN-A1u*	*-*	*+/−*	*vrn-A1u*	-//-	-//-
	TRI 17141	W	*VRN-A1u*	*-*	*+/−*	*vrn-A1u*	-//-	-//-
*T. dicoccoides* (BBAA)	IG 113301	W	*VRN-A1u’*	*-*	*+/−*	*vrn-A1u´*	KM586660	AY747598
	IG 46273	W	*VRN-A1b*	*-*	*+/−*	*VRN-A1b*	KM586654	AY616461
	IG 46306	W	*VRN-A1b*	*-*	*+/−*	*VRN-A1b*	-//-	-//-
	IG 115810	W	*VRN-A1b*	*-*	*+/−*	*VRN-A1b*	-//-	-//-
	IG 46277	S	*VRN-A1d*	*-*	*+/−*	*VRN-A1d*	KM586655	AY616462
	IG 46288	S	*VRN-A1d*	*-*	*+/−*	*VRN-A1d*	-//-	-//-
	IG 139189	S	*VRN-A1u’*	*-*	?*/+*	*VRN-A1L*	KM586658	AY747598
	IG 46297	S	*VRN-A1u’*	*-*	?*/+*	*VRN-A1L*	-//-	-//-
	k-5198	S	*VRN-A1u’*	*-*	?*/+*	*VRN-A1L*	-//-	-//-

*In all cases the promoter region was sequenced; insertion and deletions in intron 1 were determined by PCR (see Table 1).

**vrn-A1, *vrn-A1u*, *vrn-A1u´-* recessive alleles (initial structural forms); *VRN-A1-* structurally modified (mutated) forms.

**Figure 2:**
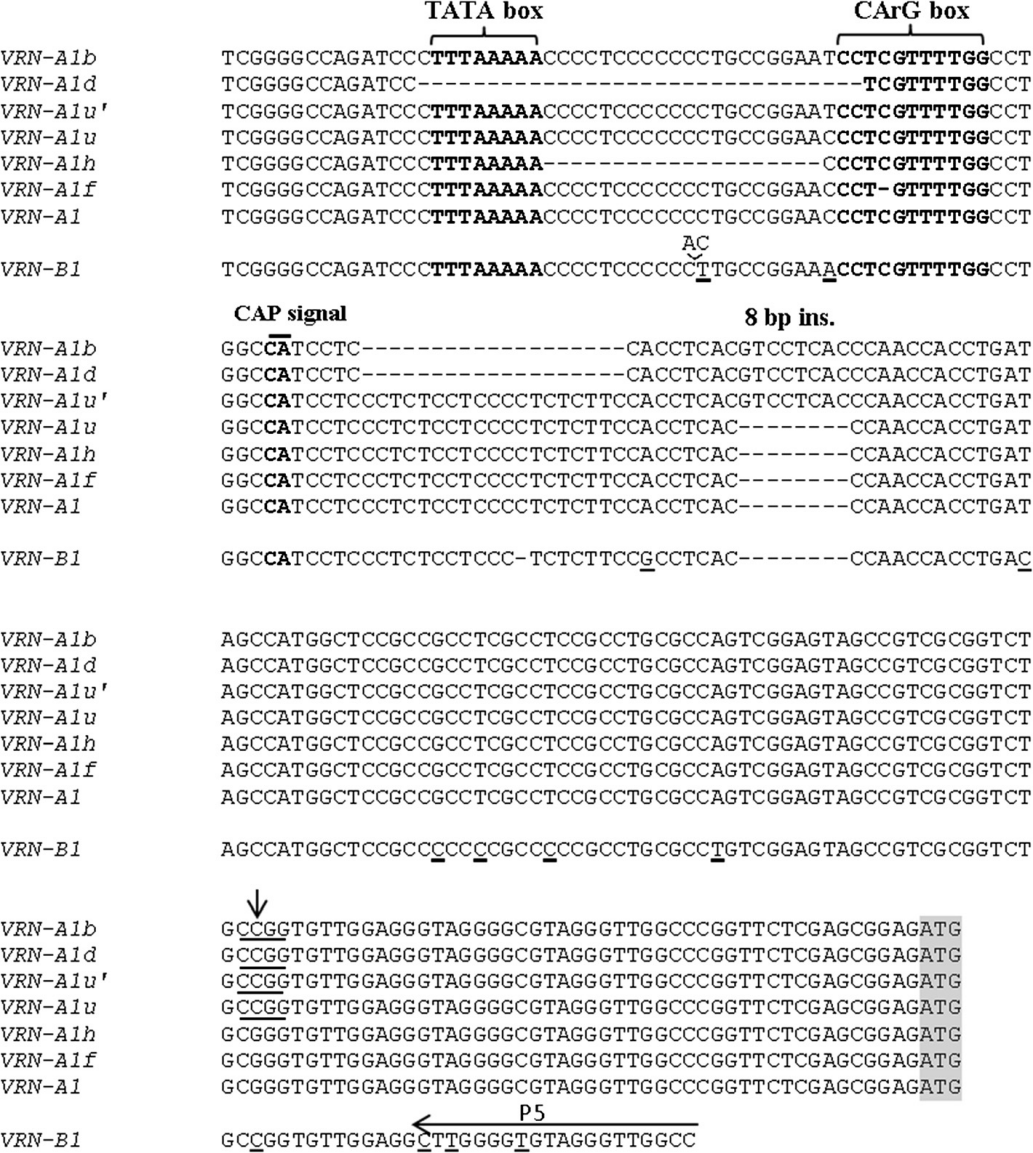
**Section (~0.2 kb) of a sequence alignment of the** ***VRN-1*** **promoter amplified with primers pairs VrnA1F/Int1R (** ***VRN-A1*** **; 0.7 kb product) and P2/P5 (** ***VRN-B1*** **; 0.8 kb product).** The location of putative transcriptional signals (in bold) are given according to Yan et al. [23]. The ATG start codon is indicated in gray. The 8 bp insertion (8 bp ins.) is shown and nucleotide substitution leading to an additional *Msp* 1 restriction site (underlined) is indicated by an arrow. Polymorphic bases in *VRN-B1* as compared to *VRN-A1* are underlined. The *VRN-B1* sequence shown here is highly conserved among five diverse accessions of *T. dicoccoides*.

**Figure 3:**
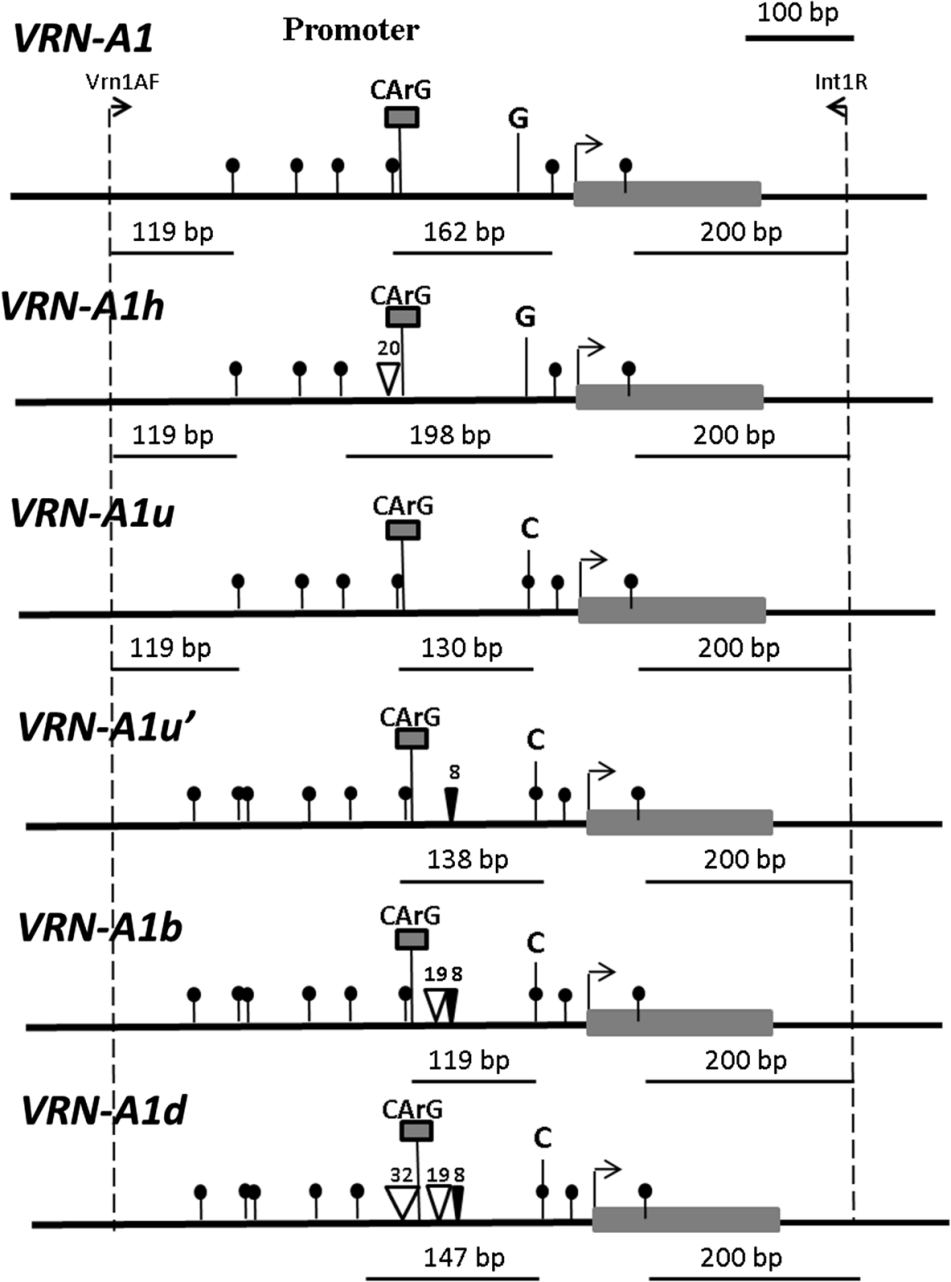
***Msp*** **I restriction enzyme map of the promoter region of different** ***VRN-1*** **alleles.** Start codon and the 1^st^ exon are indicated by the right-angled arrow flanking the grey rectangle. The positions of specific primers are shown at the top of the diagram. Dashes with black circles indicate *Msp* I restriction sites. Lengths of restriction fragments longer than 100 bp are indicated. Micro-deletions and insertions are shown by the empty and filled triangles, respectively, with sizes indicated. Positions of the CArG box and nucleotide G → C substitution are shown.

The *VRN-A1* promoter sequences of accessions IG 113301, IG 139189, IG 46297, k-5198, belonging to the first restriction pattern type (Figure 1b) were completely identical to the known sequence of the recessive *VRN-A1* allele from *T. dicoccoides* (AY747598) and contained an *Msp I* restriction fragment of 138 bp. *VRN-A1* promoter sequences of all polyploid wheats both studied here and from databases contained the 8 bp insertion and an additional *Msp* I site characteristic of the respective *VRN-A1u* allele of *T. urartu* (see below). Taking into account these features, we designated the promoter sequence of the recessive *VRN-A1* allele of *T. dicoccoides* as *VRN-A1u´* (Figures 2 and 3; Table 2; Additional file 1)*.*

Accessions IG 46273, IG 46306 and IG 115810 of the second type were identified to be a previously described *VRN-A1b* allele [23], with a 19 bp deletion in the promoter region as compared to the recessive allele (Figure 2). This deletion created the diagnostic *Msp I* restriction fragment of 119 bp (Figure 3).

Two out of 80 *T. dicoccoides* accessions (IG46277 and IG 46288) yielded a slightly smaller PCR product compared with the other accessions (Figure 1a). They were almost undistinguishable from the accessions of the first type by *Msp I* restriction (Figure 1b). Sequencing showed that these accessions contained a previously described *VRN-A1d* allele [23]. Compared with the *VRN-A1b* allele, this allele has an additional 32 bp deletion, generating a diagnostic 147 bp *Msp I* restriction fragment (Figures 2 and 3).

For analysis of the *VRN-B1* locus the primers P2/P5 were used to amplify an approximately 0.8 kb region of the promoter sequence (Table 1). No differences were revealed by PCR or subsequent *Msp* I digestion (see Additional file 2). Based on the known *VRN-B1* sequences of *T. aestivum* (AY747602-04, AY616453, AY616456), *Msp* I restriction fragments of 494, 104 and 128 bp were expected. We sequenced PCR products of 0.8 kb from 5 randomly selected accessions with diverse origins (see Additional file 1). All five sequences were highly similar to each other and to known *VRN-B1* sequences (98-100% homology). Minor variation between individual sequences was found including small deletions of up to 7 bp, however, almost all of this variation was upstream from the conserved region containing putative transcriptional signals (Figure 2). The *VRN-B1* sequences yielded in this study were deposited to Genbank under Ac.N. KM586661-65.

#### *1*^*st*^*intron*

**Figure 4:**
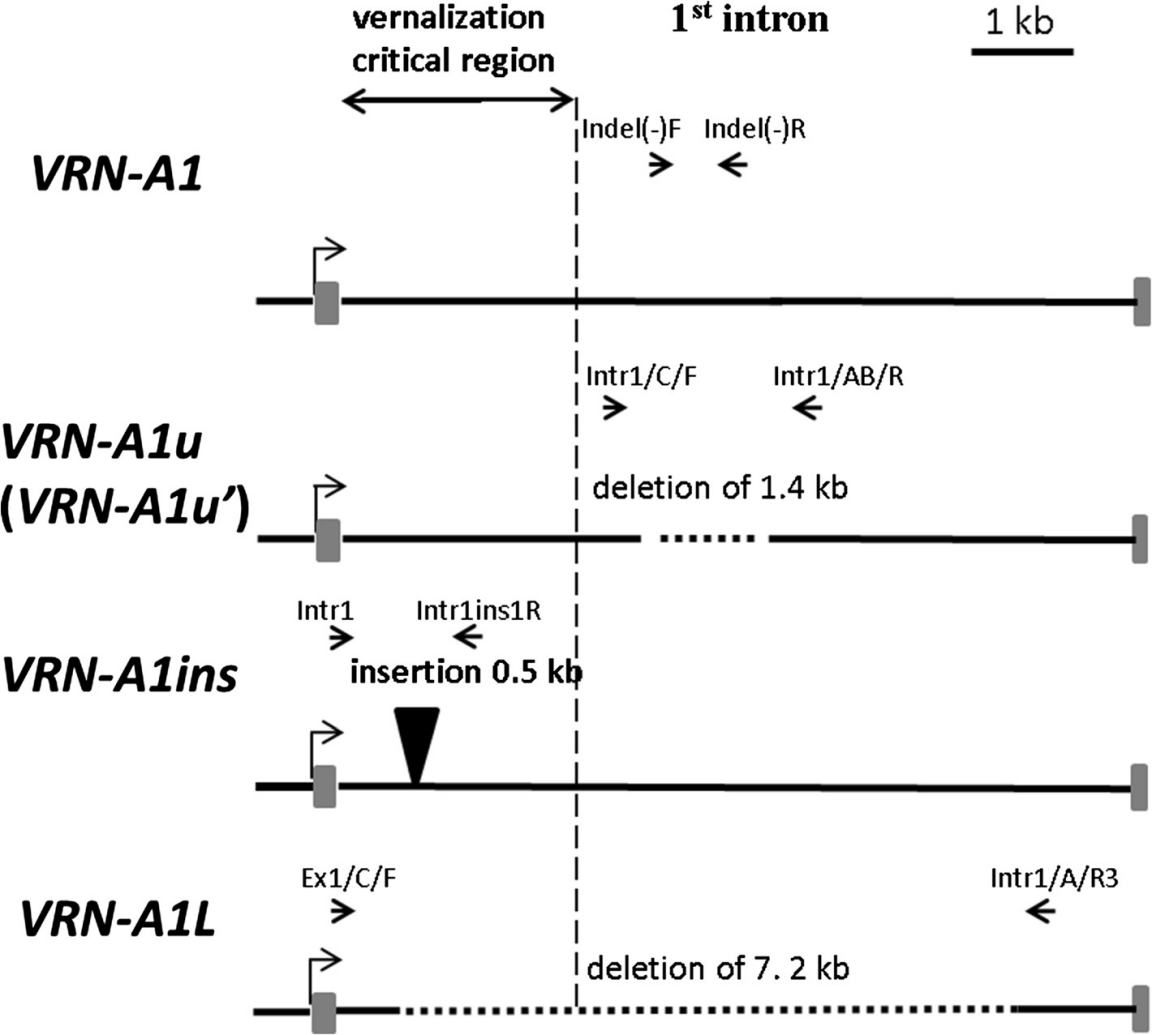
**Schematic representation of the 1** ^**st**^ **intron region of different** ***VRN-1*** **alleles.** The positions of specific primers are shown above each scheme. Large deletions and insertions are indicated.

**Figure 5:**
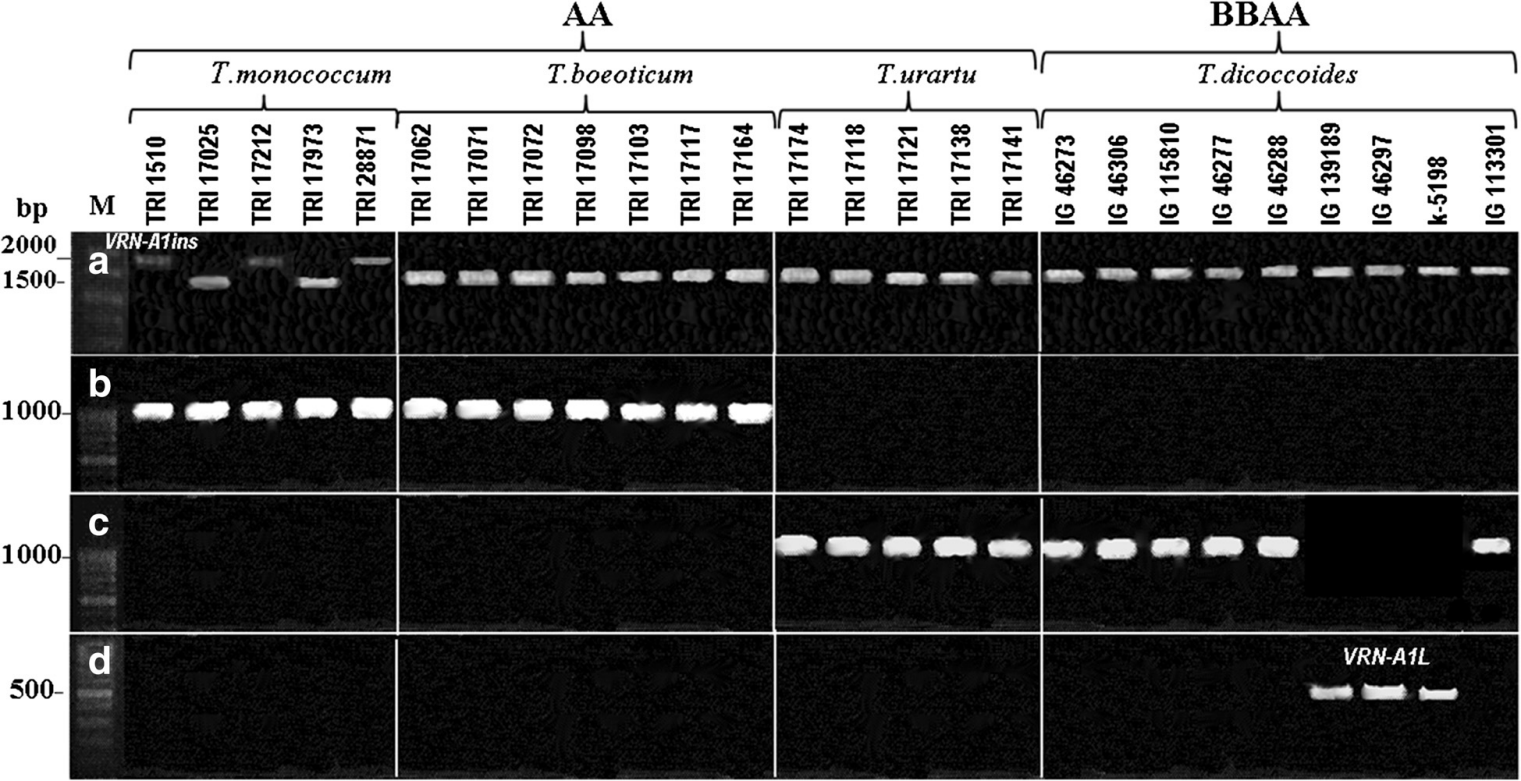
**PCR amplification of** ***VRN-1*** **intron 1 region.** PCR amplification with primers Intr1/Intr1insR **(a)**, Indel(−)F/Indel(−)R **(b)**, Intr1/C/F//Intr1/AB/R **(c)**, Ex1/C/F//Intr1/A/R3 **(d)**. Accession numbers, species and genotypes are given at the top.

The primer pair Intr1/C/F and Intr1/AB/R for the 1^st^ intron of *VRN-A1* (Table 1; Figure 4) were previously used to confirm the presence of the recessive allele of *T. aestivum* [24]. This allele contains a 1.4 kb deletion compared with the respective alleles of *T. monococcum* and *T. boeoticum* (see below). Almost all the studied accessions of *T. dicoccoides* yielded a PCR product of approximately 1 kb, indicating the presence of the deletion, except for accessions k-5198, IG 139189 and IG 46297 which gave no products (Figure 5c). Previously, *VRN-A1* allele from the tetraploid variety ‘Langdon’ (AY747598) was determined as having a 7.2 kb deletion in intron 1. Using the primers pair Ex1/C/F and Intr1/A/R3 for detection of this allele (Table 1; Figure 4), the three *T. dicoccoides* accessions generated a PCR product of about 0.5 kb (Figure 5d) identical to the corresponding sequence from ‘Langdon’. Here, we refer to this allele as *VRN-A1L* (Figure 4; Table 2)*.*

The pair of primers Intr1/B/F and Intr1/B/R4 was designed as a positive control for the absence of large mutations (deletions or insertions) in intron 1 of the *VRN-B1* locus [24] (Table 1). Using these primers, all accessions of *T. dicoccoides* yielded a 1149 bp PCR product indicating the presence of the wild recessive *VRN-B1* allele (see Additional file 2).

Thus, tetraploid *T. dicoccoides* displayed structural variation both at the promoter and 1^st^ intron regions of *VRN-A1* locus, whereas at *VRN-B1* no significant differences were found in the studied accessions. To determine whether this variation originated at the tetraploid level or in diploid donors of the A genome, we conducted an analysis of diploid progenitor species.

### *VRN-1* allelic variability in diploid *Triticum* species

#### Promoter region

We analysed *VRN-1* promoter variability in 160 accessions of the diploid wheat species *T. monococcum*, *T. boeoticum* and *T. urartu* (Additional file 1). As for the tetraploid accessions, the diploids yielded PCR products of ~0.7 kb with primers Vrn1AF and Int1R (Figure 1a). *Msp* I- digestion yielded three patterns of restriction (Figure 1b). The first pattern contained bands of 119, 162 and 200 bp, consistent with the known recessive *VRN-A1* allele of *T. monococcum* (AY188331). This pattern was characteristic of all 61 accessions of *T. monococcum* and 39 accessions of *T. boeoticum* (Additional file 1). The remaining 20 accessions of *T. boeoticum* yielded a pattern with no middle band at 162 bp, and intensive 200 bp band. All accessions of *T. urartu* were distinguished from the other two species by a double band of about 120–130 bp (Figure 1b). To further analyze allelic variation in the *VRN-A1* promoter, we selected a subset of accessions representing the three restriction patterns and sequenced the corresponding PCR products obtained with VrnA1F/Int1R primers (Table 2).

Among five selected accessions of *T. monococcum*, four had identical promoter *VRN-A1* sequences to the reported *VRN-A1* allele (AY188331) (Table 2). Accession TRI 28871 had a 1 bp deletion in a CArG-box motif in the promoter region (Figure 2). This allele has been previously identified in a spring accession of *T. monococcum* as *VRN-A1f* [16]. CAPS marker screening of all diploid accessions (see Methods) revealed a 112 bp band characteristic of *VRN-A1f* (Figure 1c) in three more accessions of *T. monococcum* (Additional file 1). Subsequent sequencing confirmed the presence of *VRN-A1f* in these accessions.

Of seven selected accessions of *T. boeoticum*, three had promoter sequences which were identical to the recessive *VRN-A1* allele of *T. monococcum* (Table 2)*.* Sequences of the remaining four accessions were identical to the previously described *VRN-A1h* allele (GQ451745), which differs from *VRN-A1* by a 20 bp deletion near the CArG- box (Figure 2). This deletion resulted in a loss of one *Msp* I site (Figure 3), explaining the absence of the 160 bp band and increased staining intensity of the 200 bp band due to superposition of a 198 bp fragment (Figure 1b). We concluded that the 20 *T. boeoticum* accessions showing this restriction pattern carry *VRN-A1h*, whereas the remaining accessions are likely to have *VRN-A1* (Additional file 1).

The five selected accessions of *T. urartu* had an identical *VRN-A1* promoter sequence (Table 2), with 98% sequence identity to the respective sequence of *T. monococcum VRN-A1*. Of the few nucleotide substitutions, one (G → C, position −48 from the start codon) resulted in a new *Msp I* restriction site (Figure 2, 3). This explains the appearance of a 130 bp band coupled with the loss of the 160 bp band (Figure 1b). Based on our restriction analysis across the three diploid species, this substitution is specific for *T. urartu*, so we have designated the corresponding allele as *VRN-A1u* (Additional file 1).

#### 1^st^ intron

Alignment of the known *VRN-A1* sequences from databases at the 1^st^ intron revealed two large indels (Figure 4). An insertion of 0.5 kb (*VRN-A1ins*) was found previously in two spring accessions of *T. monococcum* near the 5′ side of intron 1 [31]. A deletion of 1.4 kb located about 4 kb downstream from the start of intron 1 was found in all known *VRN-A1* sequences obtained from polyploid wheat species in comparison with *T. monococcum.*

We designed specific primers flanking both indels to study variation at the 1^st^ intron of *VRN-A1* in three diploid species (Table 1; Figure 4). The primers Indel(−)F and Indel(−)R allow identification of the *VRN-A1* allele of *T. monococcum* without the deletion of 1.4 kb, with an expected PCR product of approximately 1 kb. All accessions of *T. monococcum* and *T. boeoticum* yielded this product, whereas no product was obtained with the *T. urartu* accessions (Figure 5b). The primers Intr1/C/F and Intr1/AB/R flanking the 1.4 kb deletion were used as a positive amplification control for the presence of this deletion in *T. urartu* (Table 1; Figure 4). As for *T. dicoccoides* (see above), all *T. urartu* accessions yielded the 1 kb product. This product was absent for all accessions of *T. monococcum* and *T. boeoticum*, confirming the previous result (Figure 5c). Hence, the 1.4 kb deletion in intron 1 is a specific marker for the *VRN-A1u* (*VRN-A1u´*) allele of *T. urartu* and polyploid species with an A-genome (Additional file 1).

Using primer pair Intr1/Intr1insR, 19 accessions of *T. monococcum* generated a 2 kb PCR product, demonstrating that these accessions carried *VRN-A1ins* (accessions TRI 1510, 17212 in Figure 5a; additional file 1). The remaining 42 *T. monococcum* accession yielded a fragment of about 1.5 kb indicating the absence of *VRN-A1ins.* Similarly, no *VRN-A1ins* was found in any of the *T. urartu* or *T. boeoticum* accessions, excepting a single *T. boeoticum* accession from Europe (which may have been mis-classified) (Additional file 1).

### Evaluation of growth habit in diploid and tetraploid accessions

To assess the impact of *VRN-1* alleles on vernalization requirements in diploid and tetraploid wheats, we selected 26 accessions containing both initial (recessive), or structurally modified forms of *VRN-1* genes (Table 2). Noteworthy, in two accessions of *T. monococcum* and 1 accession of *T. boeoticum* the recessive form of *VRN-A1* (*VRN-A1* promoter, no insertion in intron 1) was associated with spring growth habit, implying an involvement of other genes in the determination of this habit. Four accessions of *T. monococcum* carrying a *VRN-A1f* allele was defined as a spring wheat. Out of four accessions of *T. boeoticum* carrying the *VRN-A1h* allele, two had a spring growth habit, while the other two were winter types. All five accessions of *T. urartu* with uniform *VRN-A1* sequences were winter types (Table 2).

With respect to the tetraploid *T. dicoccoides*, allele *VRN-A1b* was associated with winter growth habit in all three accessions containing this allele, whereas *VRN-A1d* and *VRN-A1L* were strongly associated with spring habit (Table 2). Accession IG 113301 with a recessive form of the *VRN-A1* gene (*VRN-A1u´* promoter, no changes in intron 1) was classified as a winter wheat.

## Discussion

Previous studies of *VRN-1* allelic variability in wild *Triticum* species were restricted either by inclusion of a limited number of accessions for analysis [16,23,24,31], or by the use of a single regulatory region (*VRN-1* promoter) as a marker [32]. Here we have made an attempt to systematically study these species using a wide set of accessions representing wild tetraploid *T. dicoccoides* and three diploid A genome progenitor species (Additional file 1).

### *VRN-1* allelic diversity in diploid A genome species

A CAPS markers designed to the promoter *VRN-1* region allowed us to reveal the three most frequent allelic variants in diploids, while avoiding a large scale sequencing approach (Figure 1). The recessive *VRN-A1* allele was prevalent among the accessions of *T. monococcum* and *T. boeoticum* (Additional file 1; Table 2). Twenty accessions of *T. boeoticum* contained allele *VRN-A1h* which was absent from all accessions of *T. monococcum.* We found the previously described *VRN-A1f* allele (Figure 2) in four accessions of *T. monococcum* (Table 2; Additional file 1)*.*

All accessions of *T. urartu* had a specific restriction pattern with the frequent base cutting enzyme, *Msp* I, allowing us to distinguish *T. urartu* from the other diploid species (Figure 1b). Sequencing determined that the corresponding *VRN-A1u* allele is highly homologous to the known *VRN-A1* alleles of polyploid wheat species. The majority of these alleles contain an 8 bp insertion which is absent in the respective sequences of diploids (Figures 2 and 3), except for a single previously studied sequence from *T. urartu* (GQ451737). We have classified the latter allele as *VRN-A1u´*, suggesting that it originated from the basic *VRN-A1u* allele of *T. urartu* and was subsequently inherited by polyploid descendants bearing an A-genome, including *T. dicoccoides* (Additional file 1)*.*

Previously, molecular markers were designed for identification of large deletions in intron 1 affecting vernalization response [24]. Using these and our own markers (Table 1) we found two major alterations occurring at different evolutionary stages of diploid wheats (Figures 4 and 5). The insertion of 0.5 kb may have occurred relatively recently during the evolution of *T. monococcum*, since it was found only in some accessions of this species (Additional file 1)*.* The deletion of 1.4 kb probably occurred in a progenitor of *T. urartu* after its divergence from *T. boeoticum*. The estimated divergence time between *T. boeoticum* and *T. urartu* and between *T. monococcum* and *T. boeoticum* is 570 000 and 290 000 YA, respectively [33]. Besides *T. dicoccoides*, the 1.4 kb deletion is characteristic of other polyploid species with an A genome*.*

Thus, the analysis of both the promoter and 1^st^ intron regions of *VRN-1* gene in diploid wheat species allowed us to reveal species-specific alleles including *VRN-A1f* and *VRN-A1ins* for *T. monococcum*, *VRN-A1h* for *T. boeoticum* and *VRN-A1u* for *T. urartu*. The structural similarity of the latter allele with the respective alleles of wheat polyploids confirmed *T. urartu* as the donor of the A genome to wheat polyploids.

### Tetraploid wheat genetic diversity at *VRN-1* loci; the beginning of the formation of a new allelic set

Wild emmer wheat *T. dicoccoides* as a species is about 360,000 years old, resulting from a spontaneous hybridization event which took place somewhere in the Fertile Crescent, a crescent-shaped area of fertile land in the Middle East [34]. *T. dicoccoides* belongs to the first cereals domesticated by humans and it is this domestication step which provided the key for subsequent bread wheat evolution [1].

Using the same approach as for the diploid wheats to study *T. dicoccoides*, we found two previously described alleles *VRN-A1b* and *VRN-A1d* with mutations in the promoter region (Figure 2 and 3; Table 2). These alleles occur in different polyploid wheats both tetra- and hexaploid but they have not been found in diploid wheat species [23,32]. We did not find the dominant *VRN-A1a* allele with insertion of a foldback element in the promoter region amongst the *T. dicoccoides* accessions (Table 1). This allele is abundant in hexaploid wheat *T. aestivum* [23,35,36].

PCR analysis of the 1^st^ intron region revealed allele *VRN-A1L* in three accessions of *T. dicoccoides* (Figure 4 and 5). This allele is common among cultivated spring varieties of tetraploid *T. durum* [24,37]. *VRN-A1L* was not found in the diploid wheat species.

Unlike *VRN-A1*, locus *VRN-B1* displayed no significant variability within either the promoter or 1^st^ intron regions (Figure 2, Additional file 2). Several large mutations, mainly deletions within the 1^st^ intron of *VRN-B1* were previously found in spring varieties of *T. aestivum* [24,25,27,35]. But these mutations have not yet been detected in tetraploid wheat species, and are thus likely to have a later origin.

The analysis of *VRN-1* polymorphism in polyploid and diploid wheat accessions showed that the two groups differ with respect to the sets of *VRN-1* alleles found among accessions.

### Effect of *VRN-1* gene structure on growth habit

#### Diploid wheat species

We determined the impact of *VRN-1* alleles on growth habit in a selected set of accessions representing three diploid species of the A genome (Table 2). Out of five tested accessions of *T. monococcum*, four were spring types despite the presence of an intact promoter region at *VRN-A1*. In two of them spring growth habit was associated with insertion in intron 1 (*VRN-A1ins*)*.* Totally, we found the *VRN-A1ins* allele in 31% of *T. monococcum* accessions (Additional file 1).

Previous data support the importance of intron 1 in the vernalization response and heading time determination, and the proposed key regulatory region has been narrowed to a highly conserved 2.8 kb “vernalization critical region” located near the left side of intron 1 [24]. A number of mutations (deletions, insertions etc.) in this region have been associated with high levels of *VRN-1* transcripts and a spring growth habit in different crops [24,26,31,38,39]. It is suggested that these mutations may affect epigenetic chromatin states, resulting in a higher basal level of *VRN-1* expression [19,26,40,41].

Dubcovsky et al. suggested that mutation in CArG- box of the *VRN-A1f* allele of *T. monococcum* results in spring habit preventing interaction with unknown repressor dependent on photoperiod [31]. However, we can not discriminate the effect of this allele on vernalization response, because in our material it always occurs in combination with *VRN-A1ins* (Table 2; Additional file 1).

In two accessions of *T. monococcum* (TRI 17025, 17973) and one accession of *T. boeoticum* (TRI 17072) spring growth habit was associated with the wild recessive *VRN-A1* allele (*VRN-A1*; no insertion in intron 1; Table 2). Alignment of *VRN-1* sequence from accessions TRI 17072 and TRI 17062 of *T. boeoticum* (spring and winter types, respectively) showed that the first accession had a mutation that has not been described previously, the substitution T → C coupled with 1 bp deletion about 200 bp downstream from the start of intron 1. It remains unclear whether this minor alteration within “critical region” (see above) is the reason for spring growth habit.

Previous studies of vernalisation genes in wheat have identified loss-of function mutations at the *VRN-2* gene, the flowering repressor, which result in up-regulation of *VRN-1* regardless of its allelic status. [15,17,31]. We studied 3 above mentioned accessions using previously developed molecular markers for *VRN-2* gene [17] and found that accession TRI 17025 of *T. monococcum* contains a mutated recessive *VRN-2* allele (data not presented). We failed to define the reason for spring growth habit in accession TRI 17973 of *T. monococcum.* In previous study, 6% of accessions of this species were of spring type despite the presence of *vrn-1/VRN-2* genotype [17]. Based on these data, it was suggested that genes other than *VRN-1* and *VRN-2* influence vernalisation response in diploid wheat.

Out of four accessions of *T. boeoticum* containing *VRN-A1h* allele and studied for vernalization requirements, two were of spring type, while the other two were associated with the winter habit (Table 2).

One plausible hypothesis has been that the spring growth habit of *T. monococcum* originated in wild populations of *T. boeoticum* and was later selected by man through cultivation [32]. However, absence of the *VRN-1h* allele in *T. monococcum* coupled with the presence of specific alleles conferring spring growth habit (*VRN-A1f*, *VRN-A1ins*) in this diploid leads us to propose an alternative hypothesis, of selection of independent mutations that appeared in *T. monococcum* during domestication.

We found no spring forms in *T. urartu* (Table 2). None of the *T. urartu* accessions included in this study had changes in the promoter or 1^st^ intron regions of *VRN-1* which might confer a spring growth habit (Additional file 1). According to Dorofeev et al. [6], all forms of *T. urartu* are winter forms. Goncharov [42] found only 2% of *T. urartu* accessions to be spring types. In another previous study, no differences in the promoter region of *VRN-1* relative to the recessive allele were found in four spring accessions of *T. urartu* [32].

#### *Tetraploid wheat*

In *T. dicoccoides* we found several *VRN-1* alleles which are widely distributed among cultivated wheat polyploids. Allele *VRN-A1b* is not always associated with spring growth habit in hexaploid wheat lines [43], in our study this allele was associated with winter habit in three accessions of *T. dicoccoides* (Table 2). Allele *VRN-A1d* influences growth habit more strongly, probably due to an additional deletion affecting the CArG box (Figures 2 and 3). It confers spring growth habit in all accessions of *T. dicoccoides* both studied here and from databases. Allele *VRN-A1L* previously found in many cultivated tetraploid forms of wheat [37] is also associated with dominant spring type (Table 2).

The question of appearance of spring growth habit in *T.diccocoides* remains open. This habit could be inherited from ancestral diploids, in particular, from ancestral form of *T.urartu*, precursor of A genome. However, as shown above, *T. urartu* is predominantly winter species. Kato et al. [44] studied geographical variation in the vernalisation requirements of *T. dicoccoides* and found that the distribution of spring forms was sporadic and restricted to warmer areas. The authors suggest that the spring type might have evolved from a previous winter prototype of *T. dicoccoides* as an adaptation to warmer conditions. Our data are consistent with this hypothesis implying that the *VRN-1* associated determinants of spring growth habit in polyploid wheat were formed after combining of diploid genomes.

## Conclusions

In the present study we investigated variability in the promoter and 1^st^ intron regions of the vernalization gene *VRN-1* in different accessions of wild tetraploid species *T. dicoccoides* and its diploid A genome progenitors. Our results indicated that a number of *VRN-1* alleles characteristic of polyploid wheats (*VRN-A1b*, *VRN-A1d*, *VRN-A1L*) are found in *T. dicoccoides*, while others (*VRN-A1a*, *VRN-B1* alleles) probably arose during later stages of polyploid evolution and were selected during domestication. The occurrence of spring forms in the diploid A genome progenitor species is only partially attributed to *VRN-1* variation, since some of these forms contained an intact recessive *VRN-1* allele, while others displayed a winter habit despite the presence of a modified *VRN-1* allele (*VRN-A1h*). The most abundant allele conferring spring growth habit in diploids was *VRN-A1ins* of *T. monococcum*, containing a 0.5 kb insertion in the 1^st^ intron. A higher variability of *VRN-1* loci in polyploid species in comparison with diploids may be explained by the hypothesis that stresses due to allopolyploidization may provoke genome instability [45]. Greater *VRN-1* loci variability gives polyploid wheat an advantage in conferring adaptation to a broader range of environments.

## Methods

### Plant material and DNA extraction

Plant material included 80 accessions of the wild tetraploid wheat species *T. dicoccoides* (BBAA) and the diploid A genome species *T. monococcum*, *T. boeoticum* and *T. urartu* (61, 59 and 40 accessions, respectively). These accessions were selected from collections in the Leibniz Institute of Plant Genetics and Crop Plant Research (IPK, Gatersleben, Germany), the ICARDA genbank (Syria) and the Vavilov All-Russian Institute of Plant Industry RAN (St Petersburg, Russia). Accession numbers, phenotypes and genotypes are given in Additional file 1.

Total genomic DNA was extracted from etiolated seedlings as described by Plaschke et al. [46] with modifications. Leaves (from 5 seeds per accession) were pooled, placed in racked collection tubes (24 per rack) and homogenised directly in extraction buffer using a FastPrep-24 (MP Biomedicals, USA).

### PCR

PCR primers reported in Yan et al. [23], Fu et al. [24], Shcherban et al. [25] were used to detect the presence of dominant or recessive alleles of *VRN-A1* and *VRN-B1* loci in diploid and polyploid wheats (Table 1). To further discriminate different alleles in promoter regions of both loci we digested corresponding PCR products with restriction endonuclease *Msp* I followed by separation of DNA fragments in 2-3% high resolution agarose gel (HydraGene Co., China). The polymorphic 1-bp deletion (*VRN-A1f*) was detected as described by Dubcovsky et al. [31]. Using primers VRN1F_F (5′-ACAGCGGCTATGCTCCAGAC-3′) and VRN1F_R (5′-GGAGGATGGCCAGGCCAAATC-3′), the second nucleotide of the reverse primer (underlined T) was mutated to generate a *Taq* I restriction site for the *vrn-A1* allele that was absent in the *VRN-A1f* allele.

The sequences of the *T. monococcum VRN-A1* gene (AY188331, DQ146421, DQ146422, DQ146423) and respective sequences of *T.aestivum* (AY747600, AY747601) were used to design specific primers flanking an insertion of ~0.5 kb and deletion of 1.4 kb in the 1^st^ intron of *VRN-A1* (Table 1; Figure 4).

PCR was performed using a DNA Thermal Cycler 480 (Perkin Elmer Cetus, USA). Reaction mixtures were in a volume of 20 μl containing 50–100 ng of genomic template DNA, 1 ng of each of primer, 0.25 mM of each dNTP, 1x reaction buffer (67 mM TrisHCl, pH 8.8; 2 mM MgCl_2_; 18 mM (NH_4_)_2_SO_4_; 0.01% Tween 20) and 1 unit *Taq* polymerase. After initial denaturation at 94°C for 2 min, 35 cycles were run at 94°C for 1 min, 55-61°C (depending on the primer pair used) for 1 min, and 72°C for 0.5-2 min, followed by a final extension at 72°C for 5 min. PCR products were separated on 1% agarose gel, stained with ethidium bromide and visualized under UV light.

### Sequencing of PCR products

Amplified DNA fragments were excised following electrophoresis, purified using a QIAquick PCR purification kit (QIAGEN, Germany) and directly sequenced using an ABI PRISM Dye Terminator Cycle Sequencing ready reaction kit (Perkin Elmer Cetus, USA) and corresponding specific primers. Sequencing was performed in an ABI PRISM 310 Genetic Analyzer (Perkin Elmer Cetus). All obtained sequences were deposited to GenBank (Table 2).

### Evaluation of growth habit

Growth habits of the diploid and tetraploid accessions containing different *VRN-A1* alleles were determined from two replicates in 2013–2014 (Table 2). Seeds were sown in the greenhouse (ICG, Novosibirsk) without vernalization under a long day photoperiod regime. Three months after sowing, when all spring standard varieties had headed, experimental materials were classified as either spring (ear emergence) or winter (no visible ear formation) types.

## Additional files

Additional file 1:
**Polymorphisms at the promoter and 1** ^**st**^ **intron regions of** ***VRN-A1*** **gene in X accessions of diploid** ***Triticum*** **species and Y accessions of tetraploid** ***T. dicoccoides.***
Additional file 2:
**Variability at** ***VRN-B1*** **locus in** ***T. dicoccoides.*** a- PCR amplification using specific primers P2/P5 to detect variation within the *VRN-B1* promoter region in different accessions of *T. dicoccoides*. b- *Msp* I restriction digestion of corresponding PCR products. c- PCR amplification with primers Intr1/B/F//Intr1/B/R4 to detect the absence of deletions in the 1^st^ intron of *VRN-B1*. Asterisks mark the accessions for which the *VRN-B1* promoter region was sequenced (Genbank: KM586661-65).
