# Supplementary figures and images for: VRN-1 gene- associated prerequisites of spring growth habit in wild tetraploid wheat T. dicoccoides and the diploid A genome species
Source: BMC Plant Biol. 2015 Mar 31;15:94. doi: 10.1186/s12870-015-0473-x (PMC4383061; doi:10.1186/s12870-015-0473-x)

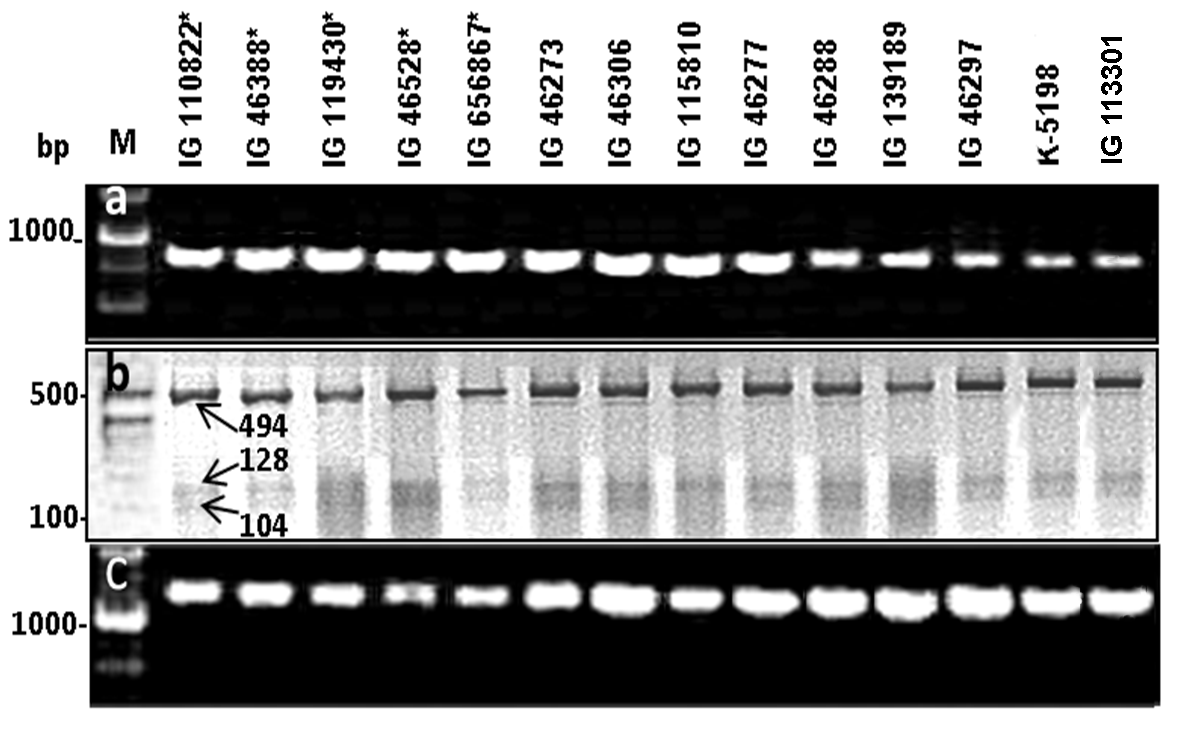

Supplement: Additional file 2: — Variability at VRN-B1 locus in T. dicoccoides. a- PCR amplification using specific primers P2/P5 to detect variation within the VRN-B1 promoter region in different accessions of T. dicoccoides. b- Msp I restriction digestion of corresponding PCR products. c- PCR amplification with primers Intr1/B/F//Intr1/B/R4 to detect the absence of deletions in the 1st intron of VRN-B1. Asterisks mark the accessions for which the VRN-B1 promoter region was sequenced (Genbank: KM586661-65). [file 12870_2015_473_MOESM2_ESM.tiff]
